# CIP2A Promotes Proliferation of Spermatogonial Progenitor Cells and Spermatogenesis in Mice

**DOI:** 10.1371/journal.pone.0033209

**Published:** 2012-03-26

**Authors:** Sami Ventelä, Christophe Côme, Juho-Antti Mäkelä, Robin M. Hobbs, Leni Mannermaa, Markku Kallajoki, Edward K. Chan, Pier Paolo Pandolfi, Jorma Toppari, Jukka Westermarck

**Affiliations:** 1 Turku Centre for Biotechnology, University of Turku and Åbo Akademi University, Turku, Finland; 2 Department of Physiology, University of Turku, Turku, Finland; 3 Department of Otorhinolaryngology, Turku University Hospital, Turku, Finland; 4 Turku Graduate School of Biomedical Sciences, University of Turku, Turku, Finland; 5 Cancer Genetics Program, Departments of Medicine and Pathology, Beth Israel Deaconess Medical Center, Harvard Medical School, Boston, Massachusetts, United States of America; 6 Department of Pathology, University of Turku, Turku, Finland; 7 Department of Oral Biology, University of Florida, Gainesville, Florida, United States of America; 8 Department of Pediatrics, Turku University Hospital, Turku, Finland; McGill University, Canada

## Abstract

Protein phosphatase 2A (PP2A) is a critical regulator of protein serine/threonine phosphorylation. However, the physiological and developmental roles of different PP2A complexes are very poorly understood. Here, we show that a newly characterized PP2A inhibitory protein CIP2A is co-expressed with ki-67 and with self-renewal protein PLZF in the spermatogonial progenitor cell (SPC) population in the testis. CIP2A and PLZF expression was shown also to correlate Ki-67 expression in human testicular spermatogonia. Functionally, CIP2A mutant mouse testes exhibited smaller number of PLZF-positive SPCs and reduced sperm counts. Moreover, seminiferous tubuli cells isolated from CIP2A mutant mice showed reduced expression of *Plzf* and other renewal genes *Oct-4* and *Nanog* at mRNA level. However, PLZF-deficient testes did not show altered CIP2A expression. Importantly, spermatogonia-specific restoration of CIP2A expression rescued PLZF expression and sperm production defects observed in CIP2A mutant mice. Taken together, these results reveal first physiological function for an emerging human oncoprotein CIP2A, and provide insights into maintenance of PLZF-positive progenitors. Moreover, demonstration that CIP2A expression can be systematically inhibited without severe consequences to normal mouse development and viability may have clinical relevance regarding targeting of oncogenic CIP2A for future cancer therapies.

## Introduction

Cellular proliferation is essential for growth of any organism as well as for normal tissue development. Moreover, animal reproduction is entirely dependent on adult germ cell proliferation. In addition to proliferation, a special mode of cellular proliferation, the self-renewal of tissue-specific stem -and progenitor cells is required both to support the renewal of tissues and for the rapid production of sperm. In testicular seminiferous tubules, the only actively proliferating and self-renewing cell types are the spermatogonial stem and progenitor cells (SPCs) that reside in the basal layer of the tubules [1]. However, the intrinsic spermatogonial genes involved in the regulation of their proliferation and self-renewal are as of yet incompletely characterized [2]. PLZF was originally identified in two mouse studies as being essential for the maintenance of the spermatogonial progenitor population and spermatogenesis [3], [4]. More recently, PLZF was specifically shown to promote spermatogonial self-renewal by opposing the function of mTORC1 [5]. However, upstream mechanisms promoting maintenance of PLZF-positive SPC pool have remained an unanswered critical question in the field [2].

Protein phosphatase 2A (PP2A) is a crucial regulator of cellular serine/threonine phosphorylation [6], [7]. Accordingly PP2A activity is essential for cellular growth and viability, and attempts to generate PP2A-targeting mouse models have been very challenging. Therefore, our current understanding of the significance of specific PP2A complex functions in mouse physiology is very limited. Recently, a novel PP2A-interacting protein designated cancerous inhibitor of PP2A (CIP2A) was shown to inhibit PP2A activity towards oncoprotein MYC [8]. CIP2A inhibition resulted in dephosphorylation of the PP2A target MYC serine 62 and consequent MYC protein degradation [8]. More recently, CIP2A's functional role as a PP2A inhibitor protein has been validated by several independent studies [9]–[11]. Moreover, proliferation defects by CIP2A depletion can be reversed by simultaneous PP2A inhibition [12]. Together these data demonstrate that CIP2A is a *bona fide* PP2A inhibitor protein. However, the physiological role of this newly discovered protein is entirely unknown so far. Here, we show that CIP2A is an important regulator of spermatogonia proliferation and spermatogenesis in mice.

## Results

### Generation of hypomorphic CIP2A^HOZ^ mice

**Figure 1:**
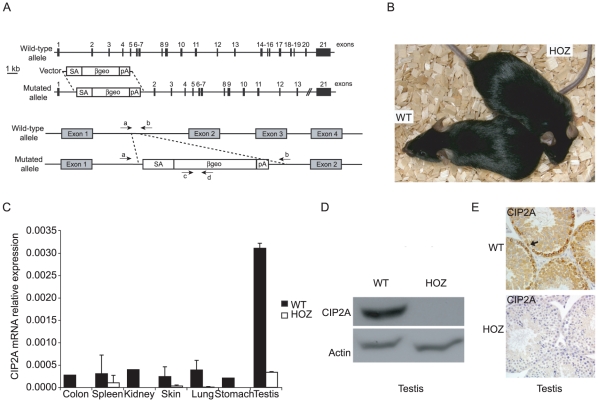
Characterization of hypomorphic CIP2A^HOZ^ mice. (A) Gene-trap strategy for inhibition of CIP2A expression. Murine CIP2A wild-type allele and the corresponding CIP2A locus with gene-trap vector insertion are presented in the upper panel (exon structure is represented with filled boxes, the gene-trap vector by clear boxes). The pGT0Lxf gene-trap vector contains a splice acceptor site (SA), β-galactosidase reporter gene (β-geo) and a SV40 polyadenylation site (pA) inserted in CIP2A locus intron 1. The lower panel indicates the position of the genotyping primers, a–b for the wild-type allele, c–d for the mutated allele. (B) CIP2A^HOZ^ mice grow and develop normally. There were no differences in morphology between adult WT or CIP2A^HOZ^ mice. (C) CIP2A mRNA expression in WT and CIP2A^HOZ^ adult mouse organs. Real-time PCR analysis for CIP2A mRNA in CIP2A expressing organs. Mouse beta-actin was used for normalization. (D) CIP2A protein expression in WT and HOZ testis tissue. (E) CIP2A immunoreactivity in WT and HOZ testis was evaluated by immunohistochemistry. No CIP2A protein expression was detected in CIP2A^HOZ^ tissues. Arrows indicate specific CIP2A staining in WT basal male germ cells.

To assess the physiological role of CIP2A, a CIP2A hypomorphic mouse model was generated using gene-trap technology. For this purpose, ES clone CD0252, containing an insertion of the pGT0Lxf gene-trap vector into the first intron of CIP2A, was derived from the International Gene Trap Consortium depository (Figure 1A). RT-PCR analysis demonstrating expression of CIP2A-LacZ cassette in CD0252 ES cells as well as genotyping result of mice homozygous for the mutated allele (CIP2A^HOZ^) are shown in figure S1. CIP2A^HOZ^ mice were viable and presented a normal lifespan of 1,5 to 2 years (data not shown), and did not show any obvious anatomical malformations (Figure 1B). CIP2A^HOZ^ mice presented a strong depletion in CIP2A expression relative to wild-type mice at mRNA level in all CIP2A-positive tissues examined (∼90%; Figure 1C). The residual expression of *CIP2A* mRNA observed in CIP2A^HOZ^ tissues was likely caused by low-level leakage of the gene-trap cassette, a phenomenon that has been reported for other cassettes [13]. In testis, loss of CIP2A protein expression in CIP2A^HOZ^ mice was verified by western blotting and immunohistochemical stainings (Figure 1D and E).

Thus, we consider the established CIP2A^HOZ^ mouse strain as a hypomorphic model suitable for assessing CIP2A's physiological role. Importantly, these results also demonstrate that CIP2A expression can be systematically inhibited without severe consequences to normal mouse development and viability.

### CIP2A expression correlates with expression of spermatogonial progenitor cell self-renewal marker PLZF and testicular germ cell proliferation

**Figure 2:**
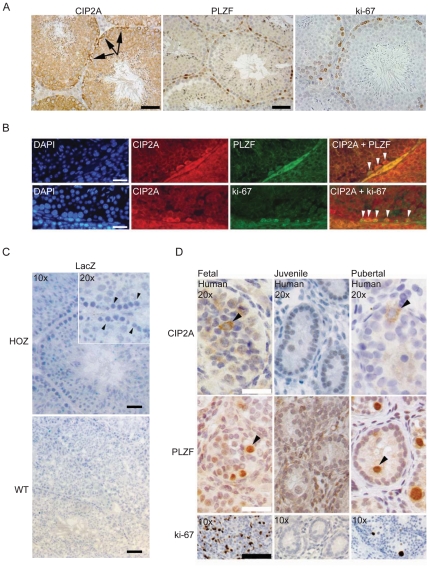
CIP2A is co-expressed with PLZF in proliferatively active primordial germ cells and spermatogonia. (A) Most primitive male germ cells, spermatogonia (indicated by arrows), locate most basally in the seminiferous tubules and contain highest CIP2A, PLZF and ki-67 expression levels in the mouse testis. (B) Immunohistochemical staining of CIP2A, PLZF and ki-67 in adult mouse testis show that CIP2A is expressed in both PLZF and ki-67 positive spermatogonia (white arrow heads). (C) CIP2A gene promoter is exclusively active in basal spermatogonia. Enzymatic LacZ staining was used to detect expression of ß-galactosidase protein from the gene-trap cassette under CIP2A promoter. ß-galactosidase activity was observed only in the CIP2A^HOZ^ spermatogonia (arrowheads in larger magnification). (D) Human testicular gonocytes (Fetal) and pubertal spermatogonia (Pubertal) express CIP2A, PLZF and ki-67 (arrowheads) whereas juvenile testis samples (Juvenile) were devoid of expression of any of these markers. White bar represents 25 µm, black bar – 50 µm.

Of all mouse and human tissue types, CIP2A is most intensively expressed in the testis (Figure 1C) [8]. Therefore, given the lack of obvious pathological consequences of systemic CIP2A inhibition, we chose to search for a potential physiological role for CIP2A in testicular biology. Anatomically, SPCs are located most basally in the seminiferous tubules, whereas their more differentiated successors, spermatocytes and spermatids, are located in the apical compartment of the tubules. Consistent with *CIP2A* and *Plzf* co-expression at all stages of the seminiferous epithelial cycle (Figure S2A), the most intense protein expression of both CIP2A and PLZF was observed in SPCs (Figure 2A). These cells also represent the only cell population that was positive for the proliferation marker ki-67 (Figure 2A). To demonstrate that CIP2A is expressed in PLZF-positive SPCs, we performed co-staining of CIP2A and PLZF by using fluorescently labeled secondary antibodies (Figure 2B). Similar strategy was employed to demonstrate that CIP2A is expressed in ki-67 positive SPCs (Figure 2B). Secondary antibody control stainings confirming the specificity of CIP2A, PLZF and ki-67 detection, and validation of these staining results also in human testis are shown in Figure S2. Based on these results, we conclude that CIP2A and PLZF are co-expressed in proliferating spermatogonial cells.

All though CIP2A protein expression is most intense in SPCs, weak staining positivity was observed also in differentiating spermatocytes (Figures 2A). To identify the testicular cell population in which *CIP2A* is transcribed, we used CIP2A^HOZ^ mice expressing the b-galactosidase protein under the control of the endogenous CIP2A promoter as an *in vivo* reporter. As shown in Figure 2C, only the basal spermatogonia, but not the differentiating spermatocytes, displayed intensive X-gal staining in the CIP2A^HOZ^ testis. The specificity of X-gal staining was confirmed by the absense of signal in the spermatogonia in the seminiferous tubules of wild-type littermates (Figure 2C). Based on these findings we conclude that *CIP2A* gene is specifically transcribed in SPCs, but the protein remains expressed also in differentiating spermatocytes. This is consistent with the reported high stability of CIP2A protein [10].

The above results demonstrate that CIP2A expression correlates with markers of progenitor self-renewal (PLZF) and proliferation (ki-67) in mouse SPC. To examine whether these findings have relevance for human physiology, we performed CIP2A, PLZF and ki-67 immunostaining on testis samples obtained from human patients of different ages. Human testicular gonocytes (precursors of spermatogonia) proliferate actively during embryogenesis, whereas the proliferation rate decreases remarkably during early childhood (juvenile). The onset of puberty again induces spermatogonial proliferation. Immunohistochemical analysis demonstrated that proliferative fetal and pubertal testes showed co-expression of CIP2A, PLZF and ki-67 proteins, whereas these markers were not expressed in the non-proliferating juvenile testis (Figure 2D, Figure S2C and Table 1). These results show that CIP2A expression co-insides with spermatogonial proliferation and PLZF positivity both in mouse and human testis.

**Table 1:** CIP2A, PLZF and ki-67 expression during human ontogenesis [(Fetal (F), Juvenile (J), Pubertal (P)], *n* = 10.

Patient no.	Age	CIP2A	PLZF	ki-67
F1	14 weeks	++	++	++
F2	17 weeks	++	++	++
F3	23 weeks	+	+	+
F4	38 weeks	−	−	−
J1	1 year	−	−	−
J2	2 year	−	−	−
J3	10 year	−	−	−
P4	12 year	++	++	+
P5	15 year	++	+	+

### CIP2A promotes spermatogenesis and maintenance of PLZF positive SPC pool

**Figure 3:**
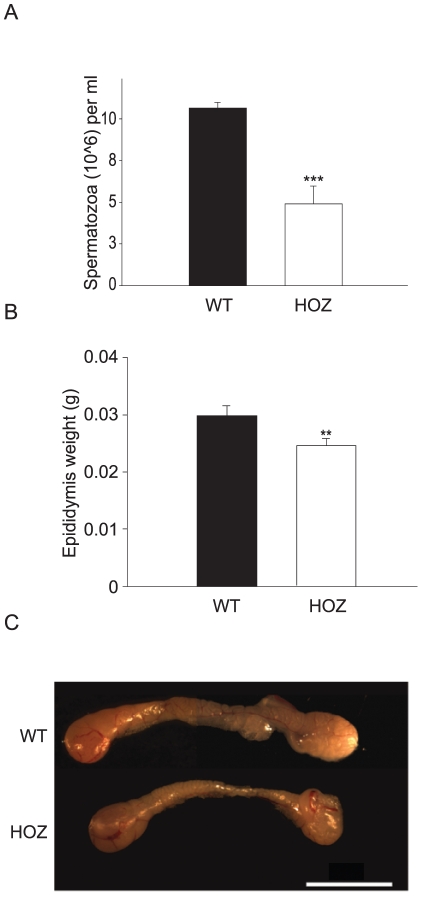
CIP2A promotes mouse spermatogenesis. (A) Decreased sperm production in CIP2A^HOZ^ mice. (*** p<0.001; n(WT) = 6, n(HOZ) = 5). (B) Decreased epididymis weight in CIP2A^HOZ^ mice. (** p<0.01, n = 6). (C) Epididymal analyses showed decreased epididymis size in CIP2AHOZ mice.

Inhibition of CIP2A did not cause any gross histological differences in testicular morphology in 8-week-old adult mice (Figure S3). However, when sperm counts of wild-type and CIP2A^HOZ^ littermates were compared, the latter showed a significant decline in sperm production (Figure 3A). In correlation with this decreased sperm production, CIP2A^HOZ^ mice also had significantly smaller and lighter epididymises (Figure 3B,C).

**Figure 4:**
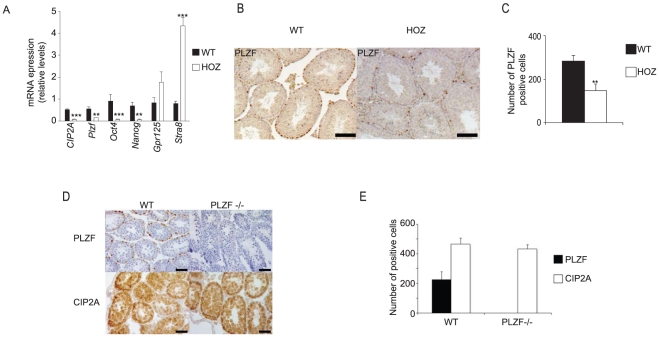
CIP2A promotes expression of spermatogonial PLZF. (A) qRT-PCR analyses showed downregulation in *CIP2A*, *Plzf*, *Oct4* and *Nanog* mRNA expression in the seminiferous tubules isolated from CIP2A^HOZ^ mice. (*** p<0.001, ** p<0.01); n(WT) = 7, n(HOZ) = 3). (B) Immunohistochemical identification of PLZF positive spermatogonia in WT and CIP2A^HOZ^ testis. (C) Quantitation of number of PLZF positive spermatogonia in WT and CIP2A^HOZ^ testis. For analysis approximately 120 tubuli per genotype were evaluated for PLZF positive spermatogonia (** p<0.01). (D) Immunohistochemical identification of PLZF and CIP2A positive spermatogonia in juvenile WT and PLZF−/− mice testis. (E) Quantitation of number of PLZF and CIP2A positive spermatogonia in WT and PLZF−/− testis. For analysis approximately 80 tubuli per genotype were evaluated. Black bar represents 50 µm.

Similarly to CIP2A^HOZ^ mice, PLZF-deficient mice show inhibition of spermatogenesis [3], [4]. Moreover, inhibition of PLZF impairs spermatogonial stem cell and progenitor self-renewal [3], [4]. This together with spermatogonia-restricted co-expression of CIP2A and PLZF prompted us to next study *Plzf* expression from isolated seminiferous tubules cells of CIP2A^HOZ^ and WT littermates. As shown in figure 4A, CIP2A inhibition resulted in significant downregulation of *Plzf* mRNA expression. Moreover, expression of other spermatogonia specific markers linked specifically with self-renewal (*Oct4*, *Nanog*) was also inhibited in CIP2A^HOZ^ mouse SPCs (Figure 4A). Importantly, while self-renewal markers were down regulated in CIP2A^HOZ^ samples, spermatogonial expression of *Gpr125*, that is not linked with self-renewal [14], or *Stra8*, a marker of spermatogonia differentiation [15], was instead increased in CIP2A^HOZ^ mice (Figure 4A). Together, these results indicate that SPCs from CIP2A^HOZ^ mice are defective in maintaining an undifferentiated state, which is analogous to recently reported phenotype of *Plzf*−/− SPCs [5].

To validate the qRT-PCR results revealing *Plzf* downregulation in CIP2A^HOZ^ samples, we performed immunohistochemical staining of PLZF on wild-type and CIP2A^HOZ^ testes (Figure 4B). Quantitation of stainings revealed significant decrease in the number of PLZF positive SPCs in CIP2A^HOZ^ testes (Figure 4C). Importantly, even though CIP2A loss inhibited PLZF positivity in SPCs, number of CIP2A positive cells was unaltered between wild-type and PLZF−/− testes (Figures 4D and E). Together, these results indicate that CIP2A functions upstream of PLZF in promoting the self-renewing progenitor identity of SPCs. Moreover, as PLZF−/− SPCs have been shown to have an impaired self-renewal capacity [3]–[5], these results suggest that CIP2A expression in SPCs is not directly reflected by the self-renewal activity of these cells.

### Spermatogonia autonomous role for CIP2A in spermatogenesis

**Figure 5:**
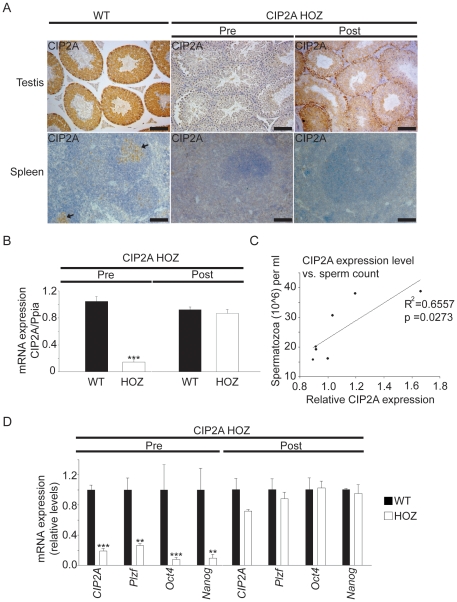
Spermatogonia autonomous recovery of CIP2A expression in CIP2AHOZ mice is sufficient to rescue sperm production and expression of self-renewal factors. (A) CIP2A staining of wild-type (WT) and CIP2AHOZ mouse testis and spleen before (Pre) and after (Post) leakage of the gene-trap cassette. CIP2A expression returned back to wild-type level in the Post-leakage testis, whereas spleen still did not show detectable CIP2A expression. Arrows in the WT spleen sample indicate CIP2A positive splenic germinal centers. (B) qRT-PCR analyses from Pre-CIP2AHOZ and Post-CIP2AHOZ seminiferous tubules demonstrated recovery of CIP2A mRNA expression in Post-CIP2AHOZ mouse testis (*** p<0.001). (C) Statistically significant correlation between CIP2A mRNA expression in the seminiferous tubules and sperm counts in a series of the Post-leakage CIP2AHOZ mice. n = 7. A representative experiment out of three independent breeding pairs displaying similar results is shown. (D) qRT-PCR analyses from the Pre-CIP2AHOZ and the Post-CIP2AHOZ mice demonstrated recovery of Plzf, Oct4 and Nanog transcription along with recovery of spermatogonial CIP2A expression in the post-leakage Post-CIP2AHOZ mice (*** p<0.001, ** p<0.01).

Results thus far show that CIP2A promotes spermatogenesis and maintenance of SPC pool. All experiments described in Figures 3 and 4 were performed with mice that were bred at most three generations after the original derivation of the CIP2A^HOZ^ mouse strain. However, when subsequent generations of the CIP2A^HOZ^ mice were analyzed for CIP2A expression, we observed a progressive increase in testicular expression of both CIP2A mRNA and protein. Intriguingly, even though CIP2A expression in the spermatogonia returned to wild-type levels in such post-leakage mice (referred as to Post-CIP2A^HOZ^)(Figure 5A,B (Post)), presence of the gene-trap insertion still reduced CIP2A expression by 90–95% in the other tissues of these mice (Figure 5A and Figure S4). We hypothesize that the observed spermatogonia-specific leakage of the CIP2A^HOZ^ gene-trap cassette may be due to alternative splicing, which can cause leakage from gene-trap cassettes [13] and is particularly prevalent in the testis [16].

The testis-specific leakage of the CIP2A^HOZ^ gene trap cassette prevented subsequent experiments directly assessing the self-renewal capacity of isolated spermatogonia. However, the natural, spermatogonia-specific reversion of CIP2A expression in Post-CIP2A^HOZ^ mice was exploited to validate the spermatogonia autonomous role for CIP2A in spermatogenesis. Importantly, by collecting mRNA samples and calculating sperm counts from a cohort of Post-CIP2A^HOZ^ mice shortly after the leakage was discovered, we were able to establish a statistically significant correlation between sperm production and CIP2A expression (Figure 5C). Similar results were observed in samples from littermates derived from two other independent breeding pairs (Figure S5). Moreover, analysis of *Plzf*, *Oct4* and *Nanog* mRNA expression in Post-CIP2A^HOZ^ spermatogonia revealed that their expression also returned to wild-type levels (Figure 5D).

**Figure 6:**
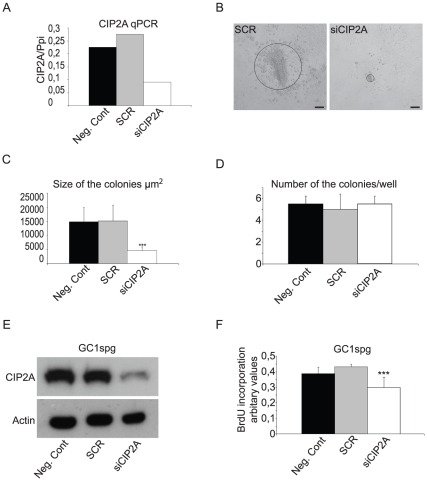
CIP2A promotes spermatogonia proliferation cell autonomously. (A) qRT-PCR analyses demonstrating reduction of CIP2A transcription in spermatogonia treated with siCIP2A. Spermatogonia were isolated from seminiferous tubules of 10-day-old mice and CIP2A mRNA expression was assessed after 7 days of CIP2A siRNA transfection. (B) siRNA-mediated downregulation of CIP2A expression inhibits colony growth of spermatogonia *in vitro*. (C) Significant inhibition of spermatogonia colony size after 7 days of CIP2A siRNA transfection. Black bar represents 100 µm. (D) CIP2A silencing does not inhibit number of spermatogonia colonies. (E) CIP2A siRNA silencing in immortalized murine spermatogonia cell line (GC1spg) show CIP2A downregulation in western blot analyses. Negative control (normal medium) and scrambled siRNA were used as a control. Beta-actin was used as a loading control. (F) Depletion of CIP2A decreased (GC1spg) proliferation significantly in vitro (*** p<0.001).

Recovery of sperm production and expression of SPC self-renewal markers by spermatogonia-specific reversion of CIP2A expression in the Post-CIP2A^HOZ^ mice strongly suggest that CIP2A supports spermatogenesis in a spermatogonia-autonomous manner. These conclusions are further supported by the unaltered levels of male sex hormones, testosterone and FSH in the circulation of CIP2A^HOZ^ mice (Table S1). To corroborate the spermatogonia-autonomous role for CIP2A by yet an independent experiment, we silenced CIP2A expression in isolated wild-type seminiferous tubuli cells by siRNA and subsequently studied the colony growth of these cells for seven days. *In vitro* culture models may not reliably mimick *in vivo* self-renewal of spermatogonia. However, *in vitro* culture experiments could be used to assess the cell autonomous role for CIP2A in spermatogonia proliferation. In isolated seminiferous tubuli cells of juvenile (under 10-day-old) mice, expressing high levels of *CIP2A*, *PLZF* and *ki-67* (Figure S6), CIP2A-specific siRNA [8] effectively inhibited CIP2A mRNA expression (Figure 6A), and caused a dramatic reduction in the size of the formed colonies (Figure 6B and C). However, CIP2A siRNA did not reduce the number of colonies formed (Figure 6D). Germ cell identity of the control cell colonies was verified by *PLZF* and *CD9* mRNA expression after 1 week *in vitro* culturing of cells (Figure S7). These data show that CIP2A promotes spermatogonial proliferation in germ cell-autonomous fashion. In line with these conclusions, CIP2A silencing also inhibited the proliferative activity of an immortalized mouse spermatogonial progenitor line (GC1spg) (Figure 6E and F).

## Discussion

Recent identification of CIP2A as a cancer-associated protein that inhibits PP2A activity towards MYC shed light on the previously unanswered question of how PP2A tumor suppressor activity is regulated in cancer cells [8]. However, the physiological function of CIP2A had not been characterized to date. Here we show that CIP2A expression correlates with the expression of spermatogonial proliferation and self-renewal markers (ki-67 and PLZF, respectively) both in human testis during ontogenesis and in mouse spermatogonia (Figure 2). The functional contribution of CIP2A to SPCs was demonstrated by the decreased sperm production and reduced expression of PLZF and other marker genes for spermatogonial self-renewal in hypomorphic CIP2A^HOZ^ mice. Interestingly, preliminary analysis of testicular phenotypes of recently generated CIP2A overexpressing mouse strain also show increased sperm counts and epididymis size (Ventelä and Westermarck, unpublished observation), further strengthening the conclusions of this work.

Spermatogonial precursors are among the most rapidly dividing cell types in the adult human body. Therefore, identifying the mechanisms that regulate proliferation and self-renewal of this cell type has been of great interest lately [2], [5], [17], [18]. PLZF has been identified as a central regulator of SPC self-renewal [3]–[5]. However, despite rapidly increasing understanding of stem cell signaling circuits, the mechanisms by which maintenance of PLZF positive SPCs is regulated have remained enigmatic [2]. The results of the current study demonstrate that CIP2A loss dramatically reduces the PLZF positivity in SPCs (Figure 4A, B, and C). Moreover, similarly to Plzf−/− SPCs [5], also CIP2A^HOZ^ SPCs seem to have increased differentiation commitment (based on increased Stra8 expression). Moreover, our data strongly indicate that CIP2A effects on proliferation are SPC autonomous. Our results also show that loss of CIP2A inhibits expression of another self-renewal protein Oct4. Interestingly, even though Oct4 is known to promote SPC self-renewal, it does not promote PLZF expression [19]. Moreover, our data show that although CIP2A does promote PLZF positivity in mouse testes, CIP2A expression is not affected by PLZF expression. These results together highlight the unique role for CIP2A upstream of PLZF among the thus far characterized SPC expressed proteins.

Taken together, results of this work give first insights into upstream mechanisms involved in maintenance of PLZF positivity in SPCs. Our results implicate a critical role for CIP2A-mediated PP2A inhibition in this process. Based on our results we propose that CIP2A's evolutionarily conserved role is to support sperm production and ensure reproduction. Therefore, it will be important to elucidate in the future whether testicular CIP2A function is altered in male infertility patients or whether CIP2A contributes to development of testicular cancers. Generally, it is expected that results of this work will have important ramifications for broader understanding of mechanisms involved in mammalian progenitor self-renewal as well as of physiological importance of ubiquitous and essential human protein phosphatase complex PP2A. Finally, demonstration that CIP2A expression can be systematically inhibited without severe consequences to normal mouse development and viability may have clinical relevance regarding targeting of oncogenic CIP2A for future cancer therapies.

## Materials and Methods

### Generation of hypomorphic CIP2AHOZ mice

The gene-trap Ola129 ES cell clone CD0252, from the Sanger Institute, presents insertion of the pGT0Lxf vector, containing the engrailed 2 gene and β-galactosidase–neomycin-resistance fusion protein (β-geo), in intron 1 of the murine CIP2A gene. The cassette expresses truncated CIP2A protein, limited to exon 1 that is fused to the β-geo protein. Expression of the CIP2A exon 1 - β-geo mRNA was confirmed by RT-PCR (Figure S1A). Chimeras were generated by using an ES cell embryo coculture method and were then mated with C57Bl6/J animals. The strain was subsequently maintained on a C57Bl6/J background. Male mice were housed in plastic cages (Tecniplast, Buguggiate, Italy) in a climate-controlled room at 21±3°C with relative humidity of 55±15% at the Animal Centre of Turku University (Turku, Finland). Aspen chips (Tapvei Co., Kaavi, Finland) were used as bedding material. Animals were maintained on a 12 h light/12 h dark cycle (lighted from 07 to 19 h) and they had free access to tap water and standard laboratory animal feed (Commercial RM3 (E) SQC, Special Diet Service, Witham, UK). All animal studies were conducted in accordance with the guidelines of the Provincial Government of Southern Finland and handled in accordance with the institutional animal care policies of the University of Turku. The Experimental Animal Committee of the University of Turku approved all protocols used in animal experiments (ESLH-2007-08517).

### Genotyping

One microgram genomic DNA from mouse ear was added to the 50 µl PCR containing 75 mM Tris HCl (pH 9.0); 50 mM KCl; 2 mM MgCl2; 20 mM (NH4)2SO4; 0.2 µM of each primer, 0.2 mM dNTPs, and 2.5 U DNA polymerase (Biotools Native DNA Polymerase, BIOTOOLS B&M Labs, S.A., Spain). The DNA was denatured at 96°C for 4 min, followed by PCR at 96 C for 45 sec, 57 C for 30 sec, and 72 C for 1 min for 25 cycles and completed by a final elongation step at 72 C for 10 min. The resulting PCR products were analyzed by electrophoresis on 2% agarose gel, and the fragments were UV visualized with ethidium bromide.

### Antibodies

Following antibodies were used for Western blotting: rabbit polyclonal anti-CIP2A [20], mouse monoclonal anti-β-actin, clone AC-74 (Sigma). The following antibodies were used for immunohistochemical stainings of paraffin-embedded tissues: CIP2A: rabbit polyclonal anti-CIP2A [20], ki-67: mouse monoclonal anti-ki67 (M7240, Dako), and PLZF: goat anti-PLZF antibody (AF2944, R&D research).

### Statistical Methods

Relative gene expression data was quantified using 2∧-DDC(t) method. The results were analyzed for statistically significant differences using one-way analysis of variance, followed by Tukey's test for multiple comparisons of independent groups of samples. The distribution of genotypes was compared to the expected Mendelian distributions with Chi-square-test. Student's t-test was used for pairwise comparisons. The p values less than 0,05 were considered statistically significant.

### Immunohisto-, cytochemistry and tissue samples

Formalin-fixed, paraffin embedded sections of mouse and human organs were cut into 6 µm thin sections, deparaffinized and thereafter rehydrated. Epitope retrieval was then proceeded in 10 mM Tris-EDTA-buffer pH 9,0 during 4 min in microwave oven 4 min 850 W followed by 15 min 150 W. After cooling down for 20 min at RT, samples were rinsed properly in water. Concerning the staining procedure, protein of the slides were firstly blocked for 10 min in 3% BSA PBS. After rinsing in Tris-HCl pH 7,4, incubation of primary antibody with CIP2A (1∶10000 rabbit polyclonal anti-CIP2A (Soo Hoo et al., 2002)), ki-67: (1∶5000 mouse monoclonal anti-ki67 (M7240, Dako)), or PLZF (1∶200 goat anti-PLZF (AF2944, R&D research)) was done for 60 min in 3% BSA/PBS or overnight in immunohistochemical staining. Control slides were incubated with normal non-immunized appropriate animal serum. Following few rinses, appropriate secondary antibody (Dako EnVision anti-rabbit or anti-mouse) was done for 30 min. Slides, again rinsed, were then incubated in DAB+ liquid Dako (K3468) for 10 min, and then rinsed in water. Samples were incubated in Mayers HTX for 1 min, rinsed with tap water, and finally dehydrated, cleared and mounted. β-galactosidase activity staining was performed as described previously (Hamalainen et al. 1999). In immunohistochemical staining the slides were incubated for 1–10 h with fluorescein-conjugated anti-rabbit, anti-Goat or anti-mouse IgG (Jackson ImmunoResearch Laboratories, Inc., West Grove, PA, USA). The usage of human tissue samples was approved by the Finnish national authority for medicolegal affairs (Dnro 889/04/047/08) and regional ethical committee of University of Turku (Dnro 146/2007). Human testicular samples were obtained from patient diagnosed testicular neoplasm and therefore underwent orchiectomy. Fetal testicular samples were collected from male fetuses underwent routine autopsy after abortion at 14–22 weeks of gestation. In according to approvals of Finnish national authority for medicolegal affairs (Dnro 889/04/047/08) and regional ethical committee of University of Turku (Dnro 146/2007) informed consent was not obtained from the participants involved in this study.

### Isolation and culture of seminiferous tubuli cells

The stages of spermatogenesis from adult mice were identified in freshly isolated mouse seminiferous tubules using a transillumination assisted identification method as described previously [21]. For in vitro cultivation seminiferous tubules from 5 mice (under 10 days old) were isolated and the cells were enzymatically separated with 0.05% trypsin for 10 min with mechanical agitation. After centrifugation seminiferous tubuli cells were cultured on 0.1% gelatine-coated culture dishes in DMEM medium containing 15% fetal calf serum and 4 ng/ml glial cell line derived neurotrophic factors (Gdnf; Tebu-Bio) and either scramble or CIP2A targeting siRNA as indicated. Medium exchange was performed on 4th day. On 7th day number and sizes of the colonies were measured and the cells were harvested for qPCR analysis. Germ cell identity of the control cell colonies was verified by *PLZF* and *CD9* mRNA expression after 1 week *in vitro* culturing of cells (Figure S7).

### Epididymal sperm count

Cauda epididymis was dissected, immersed into Dulbecco's PBS and opened by making two longitudinal and one transverse cut to allow spermatozoa to escape the epididymal tubule. After 15 min incubation at 37°C the suspension was diluted in Dulbecco's PBS and the number of spermatozoa was counted under a light microscope using the Burker chamber (Fortuna, Germany).

### Tissues sample homogenenization and RNA extraction

Liquid nitrogen frozen mice samples were homogenized using the MagNA Lyser and MagNA Lyser Green Beads. Briefly, RNA and protein samples were homogeneized in respective lysing buffers, RA1 from Macherey Nagel for RNA; RIPA buffer (1× PBS; 1% Nonidet P-40 ; 0.5% sodium deoxycholate ; 0.1% SDS) for protein samples. 1 to 4 cycles (6500 rpm, 50 sec) were used to homogeneize the tissues, an ice-cooling step of 2 min being done between each cycle. Total RNA was extracted and cleaned up from the lysate using the Nucleospin kit (Macherey Nagel), including a DNAse treatment step. cDNA synthesis and RT-PCR analysis was performed as described in supplementary protocols (Table S2).

### RT-PCR analysis

For cDNA synthesis 1 µg total RNA was incubated with 250 ng random hexamer for 5 min at 70°C, then cooled down on ice for another 5 min. Total RNA was reverse transcribed in a final volume of 25 µL containing enzyme buffer, 10 units of RNAse inhibitor, DTT, 0,5 mM deoxynucleotide triphosphate, and X units MMLV reverse transcriptase. The samples were incubated at room temperature for 10 min, then at 42°C for 50 min. The reverse transcriptase was finally inactivated by heating at 70°C for 15 min PCR amplification. The quantification was based on the standard curve method. The data were normalized using β-actin. Oligonucleotides were obtained from Proligo. For quantitative real-time PCR, 2 µL of diluted reverse transcription reaction samples (1/10) were added to 8 µL of a PCR mixture made up of 5 µL of PCR Master Mix (Applied Biosystems), 1 µL of each primer at a concentration of 3 µM, and 1 µL of specific probe at a concentration of 2 µM. The thermal cycling conditions comprised an initial step at 50°C for 2 min and a denaturation step at 95°C for 10 min followed by 40 cycles at 95°C for 15 and 60°C for 1 min. All PCRs were carried out using an ABI Prism 7000 Sequence Detection System (Applied Biosystems). The specificity of each primer couple was shown by a dissociation curve analysis. Results are derived from the average of at least two independent experiments. Gene expression was reported relative to housekeeping gene beta-actin.

### Primer sequences

Sequence information regarding all primers is presented in the table S2.

### siRNA transfections

Isolated spermatogonial cells or immortalized mouse spermatogonia (GCIspg)(ATCC CRL-2053) were adapted to 50–250 nM concentration of CIP2A [8] or scramble (SCR) siRNA or medium (negative control). siRNA was transfected with Oligofectamine reagent (Invitrogen) according to the manufacturer's instructions.

### Hormonal analyses

Serum FSH was measured by immunofluorometric assays (Delfia; Wallac, Turku, Finland) as described previously [22]. For testosterone determination serum samples were extracted twice with diethyl ether and measured using standard RIA, as described previously [23].

## Supporting Information

Figure S1 **Genotyping of hypomorphic CIP2AHOZ mice.** (A) The pGT0Lxf cassette is expressed at the mRNA level by ES cells clone CD0252. qPCR analyses of either WT CIP2A mRNA (exons 20–21 and 1–2) or pGT0Lxf cassette (exon1-LacZ) from ES cell clone CD0252 or NMuMG (Normal Murine Mammary Gland) cell line. NMuMG was used as a positive control for WT CIP2A mRNA expression and a negative control for the CIP2A exon1-pGT0Lxf transcription. B) DNA genotyping of pGT0Lxf cassette insertion into CIP2A locus at intron 1. Amplification of the wild-type (WT) allele using wild-type genomic sequence specific primers leads to a 483 bp band (left panel) whereas the mutated allele amplification product from β-geo protein specific primers is 680 bp long (right panel). Only heterozygous (het) gDNA amplify both whereas homozygous (HOZ) ones only present amplification of the genetrap cassette.
(DOC)

Figure S2 **Stage specific transcript expression of CIP2A and other spermatogonia specific markers (Plzf, Stra8).** Different stages (I–VI, VIIVIII, IX–XII) of the mouse seminiferous epithelial cycle were distinguished with transillumination-assisted identification method (Toppari et al., 1991; Ventela et al., 2000). Stage-dependent mRNA expression patterns of endogenous CIP2A and spermatogonial markers Plzf and Stra8, which are markers of spermatogonia self-renewal and differentiation, were examined (Buaas et al., 2004; Costoya et al., 2004; Zhou et al., 2008). In these analyses, Plzf and CIP2A were evenly expressed at all stages, whereas the differentiation marker Stra8 showed increased expression specifically at stages VII–VIII.
(DOC)

Figure S3 **Analyses of adult wild-type and CIP2AHOZ testes.** Haematoxylin and eosin (HE) staining showed no overt morphological differences between WT and CIP2AHOZ testicular tissues. A representative pair of organs is shown. CIP2A expression lacked in CIP2AHOZ mice.
(DOC)

Figure S4 **Inhibition of CIP2A expression in Post-CIP2AHOZ mice.** Post-leakage CIP2AHOZ mice were analyzed for CIP2A mRNA expression in tissues positive for CIP2A expression in WT mice.
(DOC)

Figure S5 **Analyses of correlation of sperm counts and CIP2A mRNA expression in seminiferous tubules from three independent breeding pairs.** (A–C) The mice originating from three independent breeding pairs demonstrated statistically significant correlation between relative CIP2A expression in the seminiferous tubules and sperm counts. Figure C is identical to Figure 4C.
(DOC)

Figure S6 **Relative CIP2A, PLZF and ki67 mRNA expression during mouse ontogenesis.** Highest CIP2A, PLZF and ki67 expression was observed in juvenile (<10 days old) testis samples.
(DOC)

Figure S7 **RT-PCR analyses of spermatogonia colony growth experiment.** Expression of spermatogonia specific marker genes in seminiferous tubulus cells cultivated for 7 days in *in vitro* conditions used for siRNA experiment shown in Figure 6.
(DOC)

Table S1 **Hormonal analyses from WT and CIP2AHOZ mice.** Plasma analyses for testosterone and FSH did not show any statistical differences between wild-type and CIP2AHOZ mice.
(DOC)

Table S2 **PCR primers and siRNA sequences used in the study.**
(DOC)
