# Supplementary material for: CIP2A Promotes Proliferation of Spermatogonial Progenitor Cells and Spermatogenesis in Mice
Source: PLoS One. 2012 Mar 26;7(3):e33209. doi: 10.1371/journal.pone.0033209 (PMC3312892; doi:10.1371/journal.pone.0033209)
Supplement: Figure S1 — Genotyping of hypomorphic CIP2AHOZ mice. (A) The pGT0Lxf cassette is expressed at the mRNA level by ES cells clone CD0252. qPCR analyses of either WT CIP2A mRNA (exons 20–21 and 1–2) or pGT0Lxf cassette (exon1-LacZ) from ES cell clone CD0252 or NMuMG (Normal Murine Mammary Gland) cell line. NMuMG was used as a positive control for WT CIP2A mRNA expression and a negative control for the CIP2A exon1-pGT0Lxf transcription. B) DNA genotyping of pGT0Lxf cassette insertion into CIP2A locus at intron 1. Amplification of the wild-type (WT) allele using wild-type genomic sequence specific primers leads to a 483 bp band (left panel) whereas the mutated allele amplification product from β-geo protein specific primers is 680 bp long (right panel). Only heterozygous (het) gDNA amplify both whereas homozygous (HOZ) ones only present amplification of the genetrap cassette. (DOC) [file pone.0033209.s001.doc]

### Supplemental data

**CIP2A promotes proliferation of spermatogonial progenitor cells and spermatogenesis in mice**

Sami Ventelä, Christophe Côme, Juho-Antti Mäkelä, Robin M. Hobbs, Leni Mannermaa, Markku Kallajoki, Edward K. Chan, Pier Paolo Pandolfi, Jorma Toppari and Jukka Westermarck

A B

**
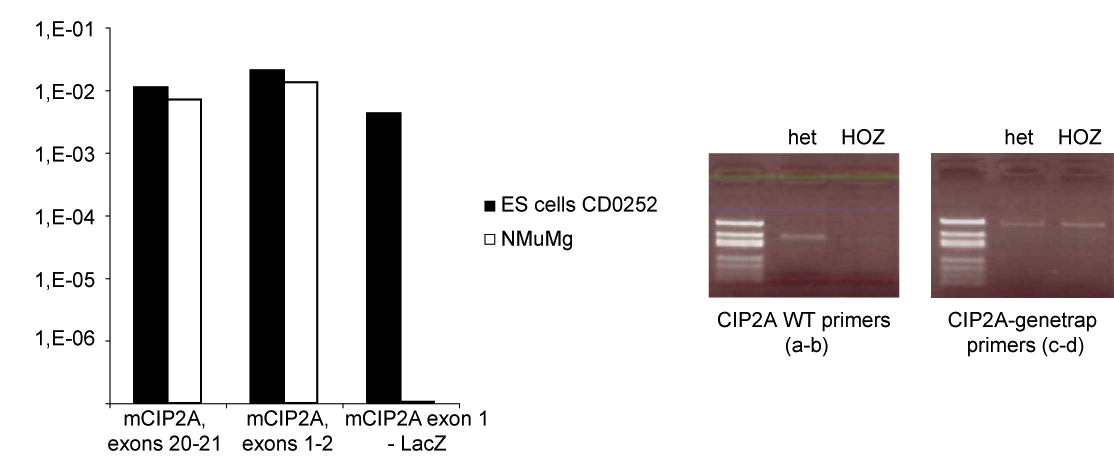
**

**Figure S1. Genotyping of hypomorphic CIP2AHOZ mice. (**A) The pGT0Lxf cassette is expressed at the mRNA level by ES cells clone CD0252. qPCR analyses of either WT CIP2A mRNA (exons 20-21 and 1-2) or pGT0Lxf cassette (exon1-LacZ) from ES cell clone CD0252 or NMuMG (Normal Murine Mammary Gland) cell line. NMuMG was used as a positive control for WT CIP2A mRNA expression and a negative control for the CIP2A exon1-pGT0Lxf transcription. B) DNA genotyping of pGT0Lxf cassette insertion into CIP2A locus at intron 1. Amplification of the wild-type (WT) allele using wild-type genomic sequence specific primers leads to a 483 bp band (left panel) whereas the mutated allele amplification product from β-geo protein specific primers is 680 bp long (right panel). Only heterozygous (het) gDNA amplify both whereas homozygous (HOZ) ones only present amplification of the genetrap cassette.
