# Supplementary material for: CIP2A Promotes Proliferation of Spermatogonial Progenitor Cells and Spermatogenesis in Mice
Source: PLoS One. 2012 Mar 26;7(3):e33209. doi: 10.1371/journal.pone.0033209 (PMC3312892; doi:10.1371/journal.pone.0033209)
Supplement: Figure S2 — Stage specific transcript expression of CIP2A and other spermatogonia specific markers (Plzf, Stra8). Different stages (I–VI, VIIVIII, IX–XII) of the mouse seminiferous epithelial cycle were distinguished with transillumination-assisted identification method (Toppari et al., 1991; Ventela et al., 2000). Stage-dependent mRNA expression patterns of endogenous CIP2A and spermatogonial markers Plzf and Stra8, which are markers of spermatogonia self-renewal and differentiation, were examined (Buaas et al., 2004; Costoya et al., 2004; Zhou et al., 2008). In these analyses, Plzf and CIP2A were evenly expressed at all stages, whereas the differentiation marker Stra8 showed increased expression specifically at stages VII–VIII. (DOC) [file pone.0033209.s002.doc]

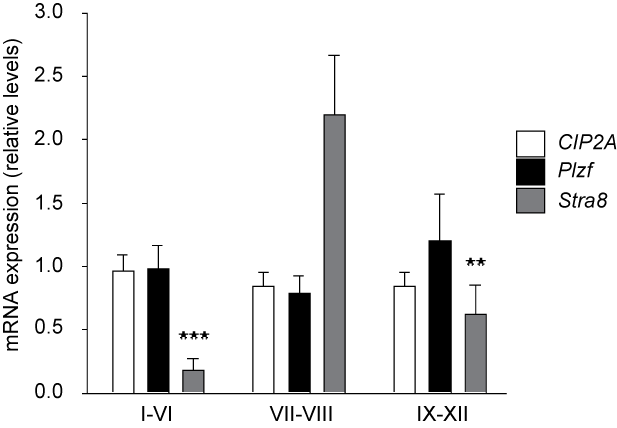
**Figure S2A. Stage specific transcript expression of CIP2A and other spermatogonia specific markers (Plzf, Stra8) were detected by qPCR.** Different stages (I-VI, VIIVIII, IX-XII) of the mouse seminiferous epithelial cycle were distinguished with transillumination-assisted identification method (Toppari et al., 1991; Ventela et al., 2000). Stage-dependent mRNA expression patterns of endogenous CIP2A and spermatogonial markers Plzf and Stra8, which are markers of spermatogonia self-renewal and differentiation, were examined (Buaas et al., 2004; Costoya et al., 2004; Zhou et al., 2008). In these analyses, Plzf and CIP2A were evenly expressed at all stages, whereas the differentiation marker Stra8 showed increased expression specifically at stages VII-VIII.

**
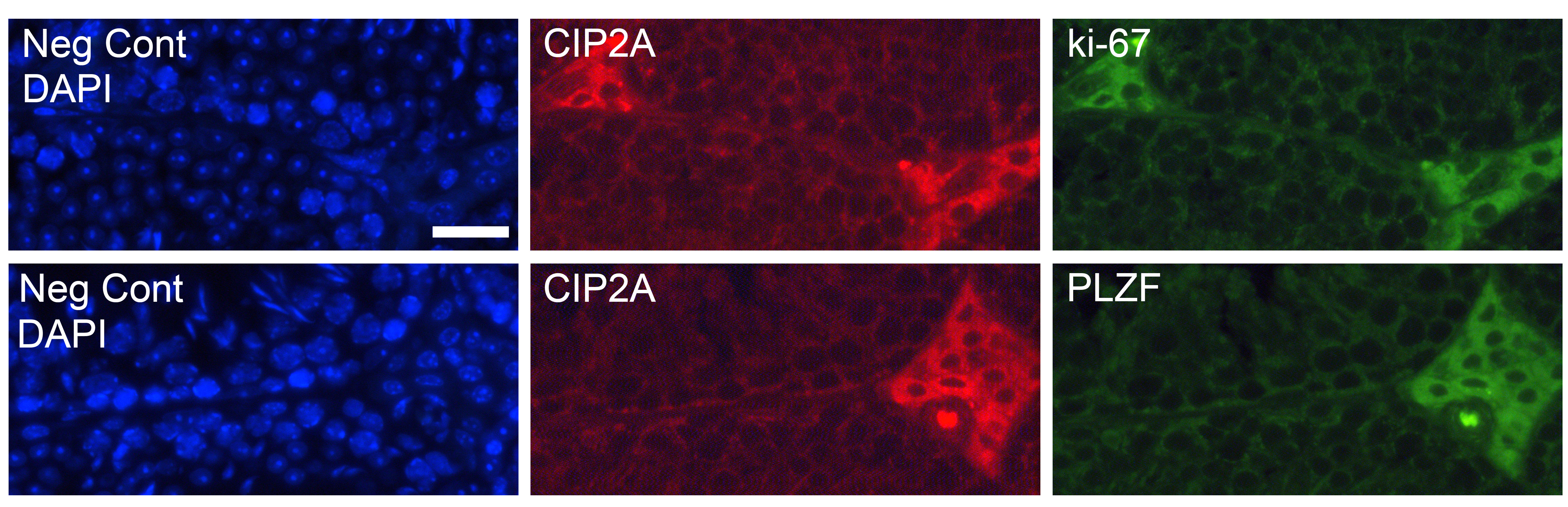
**

**Figure S2B.** Negative controls of CIP2A, PLZF and ki-67 immunohistochemical staining in adult mouse testis. Interstitial tissue gave strong red and green autofluorescence. White bar represents 25 μm.

**
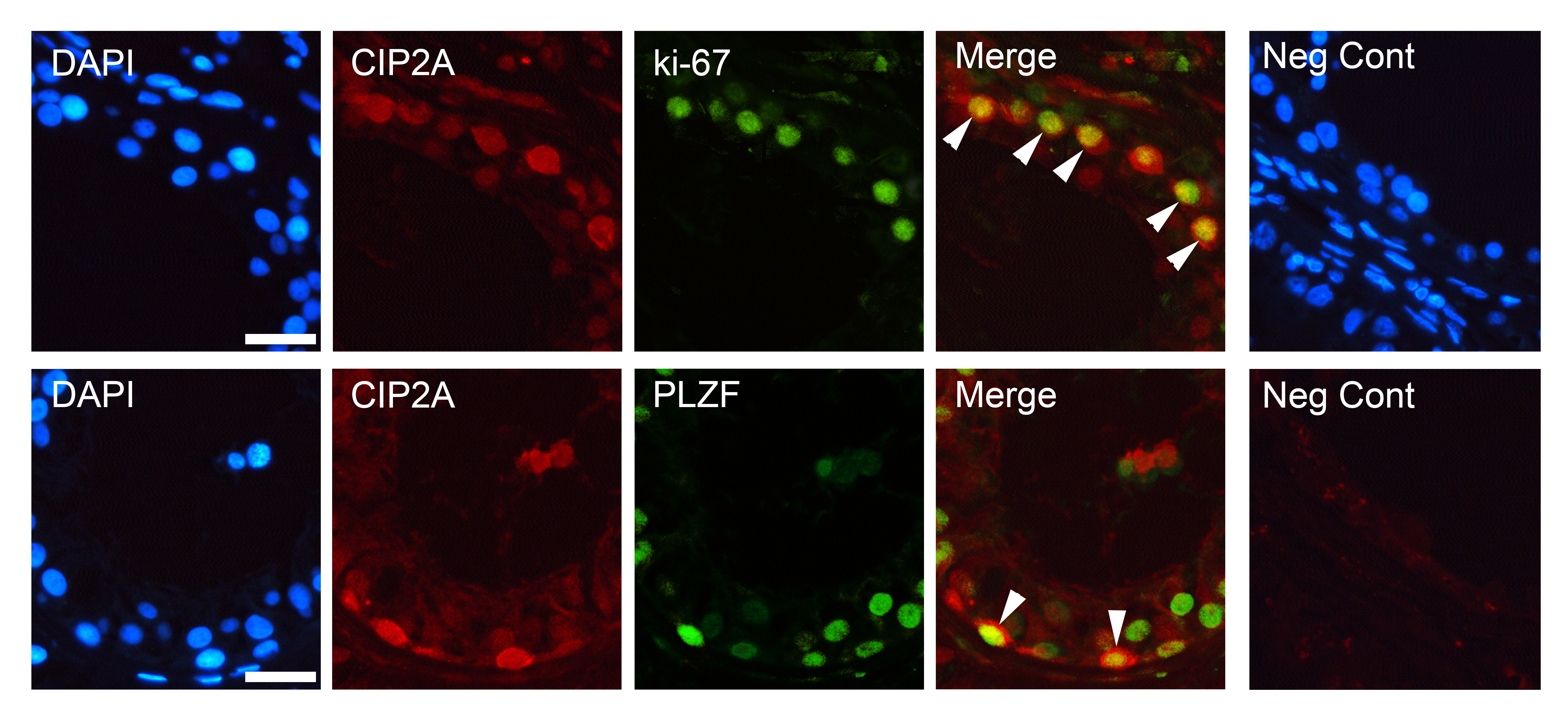
**

**Figure S2C.** Immuno-histochemical staining of CIP2A, PLZF and ki-67 in juvenile human testis show that CIP2A is expressed in both PLZF and ki-67 positive spermatogonia. Negative controls showed no immunopositivity. White bar represents 25 μm.
