# Supplementary material for: CIP2A Promotes Proliferation of Spermatogonial Progenitor Cells and Spermatogenesis in Mice
Source: PLoS One. 2012 Mar 26;7(3):e33209. doi: 10.1371/journal.pone.0033209 (PMC3312892; doi:10.1371/journal.pone.0033209)
Supplement: Figure S3 — Analyses of adult wild-type and CIP2AHOZ testes. Haematoxylin and eosin (HE) staining showed no overt morphological differences between WT and CIP2AHOZ testicular tissues. A representative pair of organs is shown. CIP2A expression lacked in CIP2AHOZ mice. (DOC) [file pone.0033209.s003.doc]

**
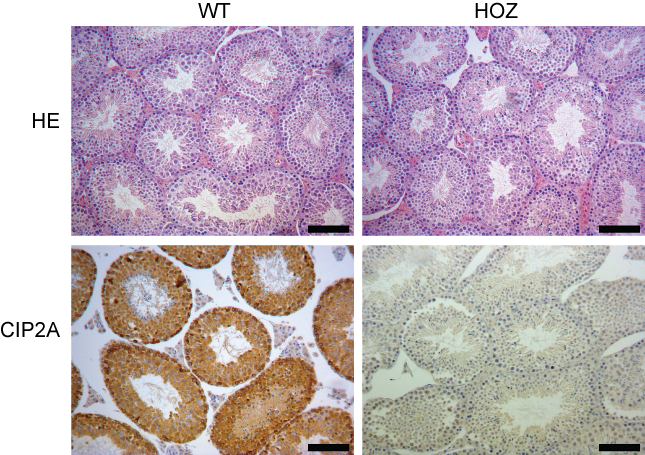
**

**Figure S3. Analyses of adult wild-type and CIP2A^HOZ^ testes.** Haematoxylin and eosin (HE) staining showed no overt morphological differences between WT and CIP2A^HOZ^ testicular tissues. A representative pair of organs is shown. CIP2A expression lacked in CIP2A^HOZ^ mice.
