# Supplementary material for: CIP2A Promotes Proliferation of Spermatogonial Progenitor Cells and Spermatogenesis in Mice
Source: PLoS One. 2012 Mar 26;7(3):e33209. doi: 10.1371/journal.pone.0033209 (PMC3312892; doi:10.1371/journal.pone.0033209)
Supplement: Figure S4 — Inhibition of CIP2A expression in Post-CIP2AHOZ mice. Post-leakage CIP2AHOZ mice were analyzed for CIP2A mRNA expression in tissues positive for CIP2A expression in WT mice. (DOC) [file pone.0033209.s004.doc]

**
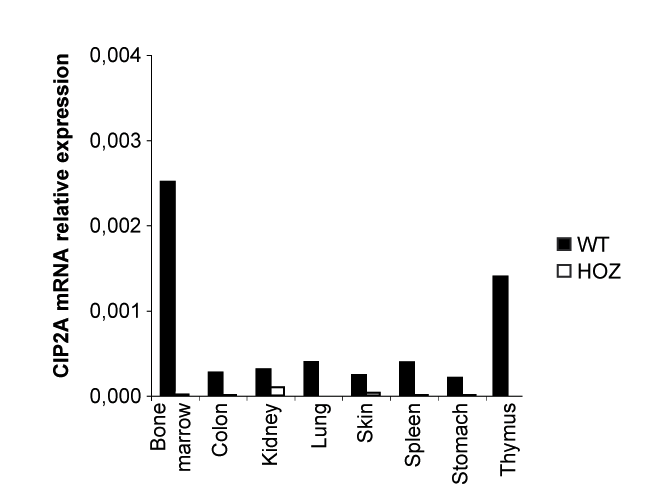
**

**Figure S4. Inhibition of CIP2A expression in Post-CIP2A^HOZ^ mice.**

Post-leakage CIP2A^HOZ^ mice were analyzed for CIP2A mRNA expression in tissues positive for CIP2A expression in WT mice.
