# Supplementary material for: CIP2A Promotes Proliferation of Spermatogonial Progenitor Cells and Spermatogenesis in Mice
Source: PLoS One. 2012 Mar 26;7(3):e33209. doi: 10.1371/journal.pone.0033209 (PMC3312892; doi:10.1371/journal.pone.0033209)
Supplement: Figure S5 — Analyses of correlation of sperm counts and CIP2A mRNA expression in seminiferous tubules from three independent breeding pairs. (A–C) The mice originating from three independent breeding pairs demonstrated statistically significant correlation between relative CIP2A expression in the seminiferous tubules and sperm counts. Figure C is identical to Figure 4C. (DOC) [file pone.0033209.s005.doc]

**
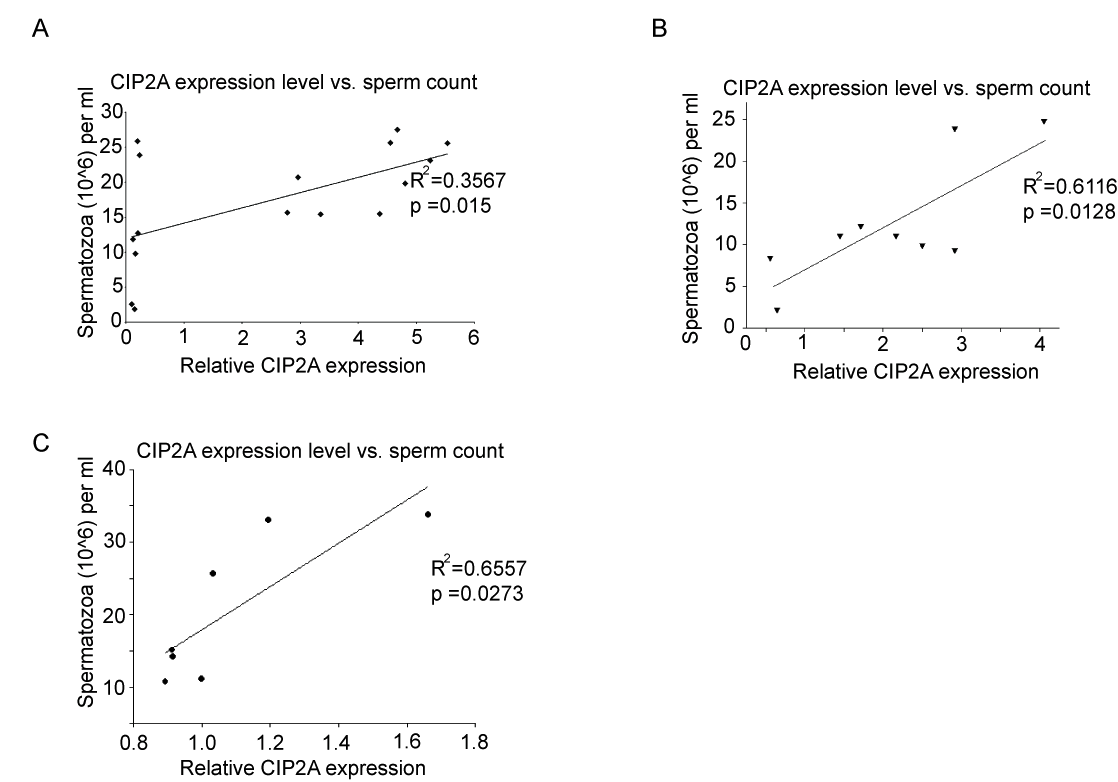
**

**Figure S5. Analyses of correlation of sperm counts and CIP2A mRNA expression in seminiferous tubules from three independent breeding pairs. (**A-C) The mice originating from three independent breeding pairs demonstrated statistically significant correlation between relative CIP2A expression in the seminiferous tubules and sperm counts. Figure C is identical to Figure 4C.
