# Supplementary material for: CIP2A Promotes Proliferation of Spermatogonial Progenitor Cells and Spermatogenesis in Mice
Source: PLoS One. 2012 Mar 26;7(3):e33209. doi: 10.1371/journal.pone.0033209 (PMC3312892; doi:10.1371/journal.pone.0033209)
Supplement: Figure S6 — Relative CIP2A, PLZF and ki67 mRNA expression during mouse ontogenesis. Highest CIP2A, PLZF and ki67 expression was observed in juvenile (<10 days old) testis samples. (DOC) [file pone.0033209.s006.doc]

**Figure S6. Relative *CIP2A*, *PLZF* and *ki67* mRNA expression during mouse ontogenesis.** Highest *CIP2A*, *PLZF* and *ki67* expression was observed in juvenile (< 10 days old) testis samples.
