# Supplementary material for: CIP2A Promotes Proliferation of Spermatogonial Progenitor Cells and Spermatogenesis in Mice
Source: PLoS One. 2012 Mar 26;7(3):e33209. doi: 10.1371/journal.pone.0033209 (PMC3312892; doi:10.1371/journal.pone.0033209)
Supplement: Figure S7 — RT-PCR analyses of spermatogonia colony growth experiment. Expression of spermatogonia specific marker genes in seminiferous tubulus cells cultivated for 7 days in in vitro conditions used for siRNA experiment shown in Figure 6. (DOC) [file pone.0033209.s007.doc]

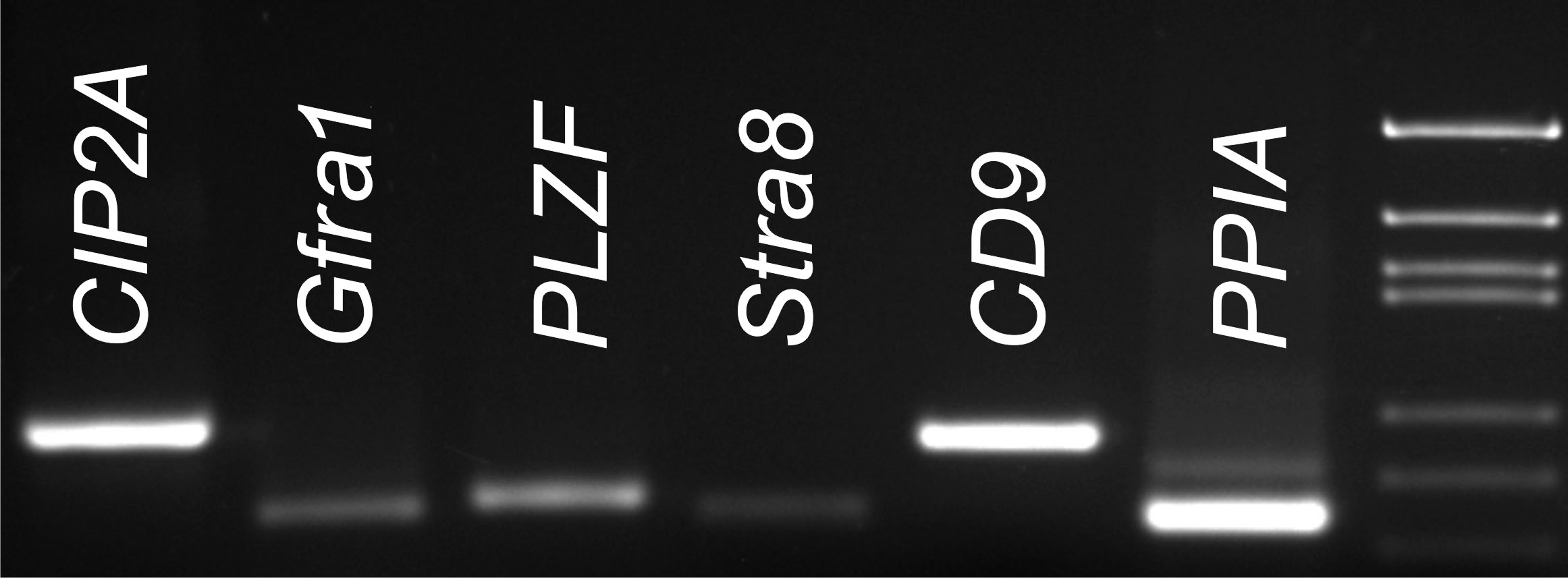


**Figure S7. RT-PCR analyses of spermatogonia colony growth experiment.** Expression of spermatogonia specific marker genes in seminiferous tubulus cells cultivated for 7 days in *in vitro* conditions used for siRNA experiment shown in Figure 6.
