# Supplementary material for: CIP2A Promotes Proliferation of Spermatogonial Progenitor Cells and Spermatogenesis in Mice
Source: PLoS One. 2012 Mar 26;7(3):e33209. doi: 10.1371/journal.pone.0033209 (PMC3312892; doi:10.1371/journal.pone.0033209)

**Table S1. Hormonal analyses from WT and CIP2AHOZ mice.** Plasma analyses for testosterone and FSH did not show any statistical differences between wild-type and CIP2AHOZ mice.


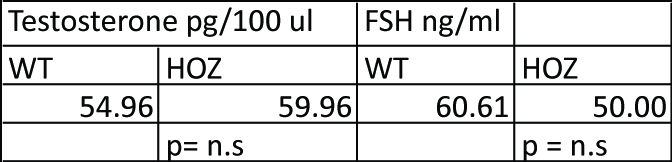

Supplement: Table S1 — Hormonal analyses from WT and CIP2AHOZ mice. Plasma analyses for testosterone and FSH did not show any statistical differences between wild-type and CIP2AHOZ mice. (DOC) [file pone.0033209.s008.doc]
