# Supplementary material for: CIP2A Promotes Proliferation of Spermatogonial Progenitor Cells and Spermatogenesis in Mice
Source: PLoS One. 2012 Mar 26;7(3):e33209. doi: 10.1371/journal.pone.0033209 (PMC3312892; doi:10.1371/journal.pone.0033209)
Supplement: Table S2 — PCR primers and siRNA sequences used in the study. (DOC) [file pone.0033209.s009.doc]

**Table S2.** PCR primers and siRNA sequences used in the study.

| **Transcript** | **Accession** | **T_annealing_** | **Primers** | **Product**  **length** |
| --- | --- | --- | --- | --- |
| *Cip2a* | NM_172616 | 57ºC | 5´-GCGCCATGTACTCAGTCAGA-3´  5´-AGGAAGCAGAAGGGTCACAA-3´ | 234 bp |
| *Gdnf* | NM_010275 | 61ºC | 5´-CGGACGGGACTCTAAGATGA-3´  5´-CGTCATCAAACTGGTCAGGA-3´ | 209 bp |
| *Gpr125* | XM_991709 | 59ºC | 5´-GACCTGACGAACAACCGAAT-3´  5´-CTGGTGTCTCGCACAGTGAT-3´ | 235 bp |
| *Ki-67* | XM_  001000692 | 56ºC | 5´-TTGGAAAGGAACCATCAAGG-3´  5´-TTTCTGCCAGTGTGCTGTTC-3´ | 214 bp |
| *L19* | NM_009078 | 55ºC | 5´-GGACAGAGTCTTGATGATCTC-3´  5´-CTGAAGGTCAAAGGGAATGTG-3´ | 195 bp |
| *Nanog* | NM_028016 | 64ºC | 5´-CCAGTGGAGTATCCCAGCAT-3´  5´-GAAGTTATGGAGCGGAGCAG-3´ | 236 bp |
| *Oct4* | NM_013633 | 64ºC | 5´-CACGAGTGGAAAGCAACTCA-3´  5´-AGATGGTGGTCTGGCTGAAC-3´ | 245 bp |
| *Plzf* | NM_  001033324 | 59ºC | 5´-AACGGTTCCTGGACAGTTG-3´  5´-CCCACACAGCAGACAGAAGA-3´ | 172 bp |
| *Ppia* | NM_008907 | 63ºC | 5´-CATCCTAAAGCATACAGGTCCTG-3´  5´-TCCATGGCTTCCACAATGTT-3´ | 164 bp |
| *Stra8* | NM_009292 | 57ºC | 5´-ATGCAATGTTGCTGAAGTGC-3´  5´-GGAAGCAGCCTTTCTCAATG-3´ | 161 bp |
|  |  |  |  |  |
|  |  |  |  |  |
|  |  |  |  |  |

| Primer | Sequence ( 5' - 3' ) | Length (bp) | %GC | probe * | probe sequence |
| --- | --- | --- | --- | --- | --- |
| mCIP2A exon 20 Fwd | GAACAGATAAGGAAAGAGTTGAGCA | 25 | 40 | 69 | CTTCCTCC |
| mCIP2A exon 21 rev | ACCTTCTAATTGAGCCTTGTGC | 22 | 45 | 69 | CTTCCTCC |
|  |  |  |  |  |  |
| m-CIP2A exon 1 Fwd | GTTAAGTCGGAGGCGAACG | 19 | 57,9 | 146 | GCTGCTGA |
| m-CIP2A exon 2 Rev | CGTGTGAGCTTTTGTCCAGA | 20 | 50 | 146 | GCTGCTGA |
|  |  |  |  |  |  |
| m-CIP2A exon 1 Fwd | GTTAAGTCGGAGGCGAACG | 19 | 57,9 | 146 | GCTGCTGA |
| pGT0Lxf 40/66 oligo | ACGCCAGGGTTTTCCCAGTCACGAC | 25 | 60 | 146 | GCTGCTGA |
| * Reference number from Roche Universal ProbeLibrary | |  |  |  |  |
